# Optimization of tomato (*Solanum lycopersicum* L.) juice fermentation process and analysis of its metabolites during fermentation

**DOI:** 10.3389/fnut.2024.1344117

**Published:** 2024-02-01

**Authors:** Lei Zhao, Ruxianguli Maimaitiyiming, Jingyang Hong, Liang Wang, Ying Mu, Bingze Liu, Huimin Zhang, Keping Chen, Aihemaitijiang Aihaiti

**Affiliations:** ^1^School of Life Science and Technology, Xinjiang University, Urumqi, China; ^2^Xinjiang Huize Food Limited Liability Company, Urumqi, China

**Keywords:** fermentation, tomato juice, superoxide dismutase, response surface methodology, UHPLC-QE-MS/MS

## Abstract

Tomato (*Solanum lycopersicum* L.) is a nutritious fruit and vegetable. Fermentation can be used to enhance their nutritional value. In this study, the tomato juice was co-fermented with multistrains, optimized by uniform experimental design and response surface methodology. Superoxide dismutase activity reached 496.67 U/g and lycopene content reached 77.12μg/g when *P. pentosaceus* (53.79%), *L. casei* (13.17%), *L. plantarum* (19.87%), *L. fermentum* (13.17%). To gain insight into the dynamics of metabolites during the tomato fermentation juice process multivariate statistical analysis was performed using the UHPLC-QE-MS/MS method. The main metabolites are peptides, amino acids carbohydrates, organic acids, and phospholipids. Carbohydrates were fully retained at the end of fermentation.The content of galactitol increased from the initial 5.389 to 6.607 while the content of cytarabine decreased by 29% and uridine by 44%. Meanwhile, phospholipids (PS, PE, PC, PG, PI) were all retained by more than 70%. Terpenoids (16-deacetylgairin, (+)-Royleanone, artemisinin) were increased to varying degrees, which gives them good nutritional value and biological activity. Organic acids (malic and citric) were reduced and lactic acid content was increased, changing its original flavor and making it more palatable to the general population. The research results have demonstrated the benefits of lactic acid bacteria fermentation on tomato juice, providing a theoretical basis and reference for the fermentation metabolism process of tomato juice.

## 1 Introduction

Tomato (*Solanum lycopersicum* L.) is a colorful, sweet and sour, nutritious fruit vegetable that is produced in large quantities globally, which is grown in almost all countries, and more than 80% are consumed as processed products (1). The global tomato production exceeded 186 million tons in 2020.^1^ Tomatoes contain a variety of bioactive substances, such as polyphenols, carotenoids, and vitamins, which have a positive impact on human health and well-being owing to their antioxidant, hypolipidemic, and anticancer properties (2). Recent studies have indicated that a quarter of the global tomato production is used for processing tomato products like sauces and ketchup, which are popular among consumers (3). The primary bioactive components in tomato products are carotenoids, particularly lycopene (LYC), which has been extensively utilized in the food industry as a functional ingredient in food supplements or new food products (4). Moreover, LYC has been shown to significantly contribute to cancer prevention and the management of chronic diseases in various pathological studies (5, 6). Although tomato products have been demonstrated to contain health-promoting nutrients and micronutrients, the research on tomato products mostly focuses on tomato sauces and ketchup, while there are few reports on deep-processing products derived from tomatoes (7).

Fermentation is a process that utilizes the growth and metabolic activities of microorganisms to stabilize and transform biomass. Beneficial metabolites, including extracellular polysaccharides, bioactive peptides, and lactic acid, can be generated by microorganisms in food matrices throughout the process of fermentation (8). Besides, fermentation techniques can effectively release bound bioactive compounds. Lactic acid bacteria (LAB) are widely used to ferment vegetables, dairy products, meats, and grains with bioactivities and a variety of health-promoting effects, including immunomodulatory, antiallergic, antio-besity, and antioxidant effects (9–11). The fermentation of fruit and vegetable juices by LAB not only preserves the nutrients and flavor of the juices but also generates bioactive substances through microbial metabolism. Compared to other strains, *Lactobacillus paracasei* fermentation of fermented orange juice was found to produce better flavor and sensory attributes as well as increased antioxidant activity (12). Furthermore, the bioactive compounds significantly increased in LAB-fermented Chinese wolfberry juice, particularly when using *Lactobacillus paracasei* and *Lactobacillus acidophilus* (13). Among LAB- fermented tomato juice (FTJ), *Lactobacillus plantarum* and *Lactobacillus casei* were found to be more suitable for developing FTJ with favorable flavor and health benefits (14). To address the challenges of tomato storage and diversify tomato products, this study aimed to develop a high-nutritional-value FTJ product.

In recent years, metabolomics has been applied to elucidate metabolite changes and biotransformation during food processes (15). UHPLC-QE-MS/MS(LC–MS) was widely used in many fields (e.g., medical research, environmental analysis, and food analysis) because of its high separation ability for complicated samples, relatively short and high sensitivity, and its ability to obtain rich structural information and molecular weight of each component. Food samples typically contain many small molecular weight metabolites that span a wide dynamic range of concentration and polarities (16). Therefore, LC–MS was chosen for its high sensitivity and resolution to analyze metabolites during the fermentation of tomato juice (17).

Currently, there is a paucity of information on the FTJ with LAB to enhance its bioactive function and its use of LC–MS for the analysis of non-volatile metabolites during FTJ. To the best of our knowledge, mixed LAB fermentation can produce a higher abundance of metabolites than single- strain LAB fermentation (18). To maximize FTJ’s mixed bacteria fermentation mechanism, it is essential to choose appropriate dominant strains that promote it, as each strain possesses unique traits and substrate adaptations. Therefore, this study was conducted to analyze the bioactive functions of LAB- FTJ and to examine the non-volatile metabolites during fermentation.

The objectives of this study were (1) Firstly, four strains with high Superoxide dismutase (SOD) activity and high LYC content were screened by a one-way test. The process was optimized by using homogeneous setup experiments and response surface methodology (RSM) to maximize SOD activity and LYC content, to improve bioaccessibility. (2) Samples were collected under different fermentation times (0, 10, 16, 20, and 22 h). Changes in chemical composition over time were traced by LC–MS analysis. The dynamic changes of carbohydrates (galactitol, aglycone, uridine), phospholipids (PS, PE, PC, PG, PI), organic acids (malic, citric, lactic), and terpenoids ((+)-Royleanone, 16-deacetylgalloylgalloylene, and artemisinin) were analyzed throughout the fermentation process. Overall, this study provides valuable new information on the benefits of fermentation in enhancing the potential nutritional value of tomatoes and other products.

## 2 Materials and methods

### 2.1 Materials

Tomato Sauce were provided by Xinjiang University (Xinjiang, China). Methanol, toluene, from Tianjin Shengao Chemical Reagent Co., Ltd. (Tianjin, China). MRS from Hangzhou Best Biotechnology Co, Ltd. (Hangzhou, China). *Lactobacillus fermentum* (*L. fermentum*) CICC 21800*, Lactobacillus acidophilus* (*L. acidophilus*) CICC 6085, *Lactobacillus reuteri* (*L. reuteri*) CICC 6126*, Lactobacillus plantarum* (*L. plantarum*) CICC 21797, *Lactobacillus paracasei* (*L. paracasei*) CICC 22709, *Lactobacillus casei* (*L. casei*) CICC 6114, *Lactobacillus delbrueckii subsp bulgaricus* (*L. bulgaricus*) CICC 20247, *Pediococcus pentosaceus* (*P. pentosaceus*) CICC 21862, *Pediococcus acidilacriti* (*P. acidilacriti*) CICC 20720, *Lactobacillus rhamnosus* (*L. rhamnosus*) CICC 6135 were obtained from the China Center of Industrial Culture Collection (CICC) (Beijing, China). Pectinase, cellulase, and hemicellulase were purchased from Fibrochem Biochemicals Co., Ltd. (Shanghai, China). All strains were activated twice and incubated at 37°C for 24 h before use. Methanol and acetonitrile were supplied by Meguiar’s. The SOD kit was purchased from Beijing Solebo Technology Co., Ltd. (Beijing, China).

### 2.2 Preparation and fermentation of tomato juice

First, tomato paste was mixed well with water 1:2.6. The Total Soluble Solid (TSS) was adjusted to 12.5^°^Brix, Then, sterilized in water at 90°C for 10 min and cooled down subsequently. Add 1.5% (w/v) pectinase, 1% (w/v) cellulase, and 1% (w/v) hemicellulase in the sample and enzymatically digest for 3 h at 57°C. Following, the samples were sterilized at 90°C for 30 min. After cooling, the strains were accessed into samples and placed under a Constant temperature oscillation incubator (ZD-85A, Changzhou, China, Setting 37°C) for 22 h of fermentation.

### 2.3 Screening of dominant strains and determination of their optimal proportions

Ten strains (*L. fermentum, L. acidophilus, L. reuteri, L. plantarum*, *L. paracasei*, *L. casei, L. bulgaricus*, *P. pentosaceus*, *P. acidilacriti*, *L. rhamnosus*) of fermented tomato homogenate were used and fermented in an incubator at 37°C for 22 h. After fermentation, the best four dominant strains were selected based on pH, SOD, LYC, TSS and sensory scores. The optimal ratio of these 4 strains were determined by four factors, a10-level, uniform design table. The results were analyzed and calculated through SPSS 26 and Excel (Table 1).

**Table 1:** Uniform design table for the experiment.

No.	*Pediococcus pentosaceus* (%)	*Lactobacillus casei* (%)	*Lactobacillus plantarum* (%)	*Lactobacillus fermentum* (%)
1	40	13	19	21
2	41	15	24	19
3	42	17	18	17
4	43	19	23	15
5	44	21	17	13
6	45	12	22	22
7	46	14	16	20
8	47	16	21	18
9	48	18	15	16
10	49	20	20	14

### 2.4 Optimization of the fermentation process

After determining the fermentation strain category and ratio, the SOD activity value and LYC value obtained after fermentation were used as indicators. The one-way experimental conditions were set as follows Soluble solids content (10.5, 11.5, 12.5, 13.5, 14.5°Brix), Inoculum volume (1.2.3.4.5*10^6^ CFU/mL), Fermentation temperature (27.32.37.42.47°C), Fermentation time (14.18.22.26.30 h), with the above four factors as the independent variables, and SOD (Y1) and LYC (Y2) as the response values, to design a Box–Behnken test. Each factor has 3 levels (−1, 0, 1) and the design includes 5 centroids for a total of 29 sets of experiments. The factors and levels of Box–Behnken are tabulated in Table 2. Response surface results are shown in Table 3.

**Table 2:** Factors and levels of the experiment.

Factors	Levels		
	−1	0	1
Fermentation temperature (°C)	32	37	47
Fermentation time (h)	18	22	26
Inoculum volume (*10^6^ CFU/mL)	2.0	3.0	4.0
Soluble solid content (^°^Brix)	11.5	12.5	13.5

**Table 3:** The experiments and results of RSM for the FTJ fermentation process.

No.	Factors				Response	
	Temperature (°C)	Time (h)	Inoculation amount (*10 ^6^CFU/mL)	Soluble solid content (^°^Brix)	SOD activity (U/mL)	LYC (μg/g)
1	37	18	2	12.5	446.09	72.01
2	37	22	3	12.5	507.71	77.69
3	37	26	3	11.5	453.06	73.63
4	42	22	3	13.5	434.46	74.71
5	42	22	3	11.5	449.24	74.17
6	32	22	2	12.5	463.62	71.19
7	32	22	2	12.5	453.18	71.46
8	37	26	4	12.5	441.22	73.63
9	42	18	3	12.5	442.63	70.92
10	37	22	2	12.5	431.77	74.44
11	42	26	3	12.5	439.04	72.01
12	37	26	3	13.5	459.96	75.80
13	37	22	3	12.5	508.07	77.97
14	37	18	3	11.5	467.97	74.99
15	42	18	3	12.5	455.31	73.91
16	32	22	4	12.5	432.41	73.91
17	37	22	3	12.5	501.41	77.43
18	42	22	4	12.5	440.63	73.10
19	38	26	2	12.5	460.23	75.80
20	32	26	3	12.5	461.62	71.74
21	37	18	3	13.5	432.72	74.18
22	37	18	4	12.5	451.92	76.09
23	37	22	3	12.5	509.67	77.17
24	37	22	2	11.5	457.04	73.65
25	37	22	3	12.5	499.37	76.63
26	37	22	4	13.5	432.67	77.70
27	42	22	2	12.5	447.08	74.45
28	37	22	4	11.5	437.18	74.72
29	32	22	3	11.5	438.09	72.83

### 2.5 Measurement of physicochemical indicators

The activity of SOD was determined according to the instructions of the kit. LYC was determined using the national standard method. The soluble solid solids content was determined using a digital refractometer (TD-45, Zhejiang, China). pH was determined by a digital desktop acidimeter (PHS-3C, Shanghai, China). The sensory evaluation was performed on the fermented samples. Samples of the fermentation broth produced under different conditions were numbered, and then 10 people trained in the sensory evaluation were asked to rate the fermentation broth product’s sensory, flavor, acceptability, and hypothetical purchase intent for a total score of 90. The details were shown in Table 4.

**Table 4:** Sensory attributes of the FTJ products.

Category	Attributes	Score
Appearance	Red color	0–10
	Clarity	0–10
Flavor	Tomato flavor	0–10
	Sour flavor	0–10
Taste	Acid-sugar ratio	0–10
	Tomato taste	0–10
	Astringency	0–10
Overall	Acceptability	0–10
	Purchase intention	0–10

### 2.6 Metabolites extraction and LC–MS analysis

Each sample (200 μL) was mixed with 800 μL of extraction solution (methanol: acetonitrile = 1:1, containing an isotope-labeled internal standard mixture) in an EP tube. The samples were then vortexed for 30 s, extracted by low-temperature ultrasound for 30 min (5°C, 40 KHz), and allowed to stand for 30 min at −20°C. The samples were then centrifuged again for 15 min (13,000 g, 4°C), and the supernatant was dried with nitrogen, followed by the addition of 120 μL of the compound solution (acetonitrile: water = 1:1). The sample was vortexed again for 30s, extracted by low-temperature ultrasonic extraction for 5 min (5°C, 40 KHz), centrifuged for 10 min (13,000 g, 4°C), and the supernatant was put into vials to be analyzed on the machine. In addition, 20 μL of supernatant from each sample was mixed as quality control (QC).

Metabolite detection was performed on an ACQUITY UPLC HSST3 column (100 mm × 2.1 mm i.d., 1.8 μm, Waters, Milford, USA) with two mobile phases: 95% ultrapure water +5% acetonitrile (containing 0.1% formic acid) and 5% ultrapure water +47.5% acetonitrile +47.5% isopropanol +5% ultrapure water (containing 0.1% formic acid). The two mobile phases were 0.1% formic acid, the temperature of the autosampler was 40°C, and the injection volume was 3 μL. The ion source was electrospray ionization (ESI), and the ionization modes: positive (ESI+) and negative (ESI-) scanning modes were used to collect mass spectrometry signals. The scanning range was 70 ~ 1,050 m/z; the intrathecal gas flow rate was 50 Arb, the auxiliary gas flow rate was 13 Arb, and the capillary temperature was 325°C. The resolution of the full mass spectrum was 60,000, and the resolution of the MS2 was 7,500. The collision energies were 20, 40, and 60 eV.

### 2.7 Data processing and statistical analysis

Data were expressed as mean plus or minus standard deviation and analyzed using SPSS26 software [In SPSS 26, we use the one-way ANOVA test to compare means. Statistical significance (*p* < 0.05; Duncan test)]. Plotting was done using Origin 2021 software. Multivariate analysis was performed using SIMCA 16.0 software. Principal Component Analysis (PCA) and Partial Least Squares Discriminant Analysis (PLS-DA) were performed to reduce the dimensionality of the data by visualizing sample distribution and grouping. Subsequently, supervised Orthogonal Projection-Least Squares Discriminant and Applied Analysis (OPLS-DA) of the underlying structure was performed, and additionally, to assess the importance of the variables in the projection, the importance of the projected variables of the initial principal components (VIP) was determined in Orthogonal Partial Least Squares Discriminant Analysis (OPLS-DA). This technique allows for summarizing and determining the contribution of each variable. Metabolites with VIP > 1 and *p* < 0.05 were considered as significantly changed metabolites.

## 3 Results

### 3.1 Strain screening and ratio determination

Each strain has its characteristics during the fermentation process, so it is important to consider the characteristics of each strain when selecting the appropriate strain for tomato juice. The effect of multi- strain association on fermentation is more beneficial than single-train fermentation due to the reciprocal symbiotic interaction between microorganisms and products derived from mixed fermentation containing more metabolites (19). In this experiment, 10 strains were used for fermentation, SOD, LYC, pH, TSS, and sensory during fermentation were determined, and finally four strains were selected for mixed fermentation. The results were shown in Table 5.

**Table 5:** Physicochemical properties of FTJ fermented by ten strains.

No.	Strains	SOD activity (U/g)	LYC (μg/g)	pH	TSS	Sensory Evaluation (Score)
1	Unfermented	387.00 ± 2.00^e^	68.75 ± 1.09^de^	3.98 ± 0.03^cd^	13.80 ± 0.10^cde^	83.33 ± 1.52^cd^
2	*P. pentosaceus*	420.47 ± 3.01^c^	75.43 ± 1.18^a^	4.02 ± 0.00^ab^	14.20 ± 0.10^ab^	88.33 ± 1.52^ab^
3	*L. casei*	428.44 ± 1.66^b^	72.24 ± 0.71^bc^	4.02 ± 0.00^ab^	14.10 ± 0.26^ab^	85.00 ± 1.73^bcd^
4	*L. rhamnosus*	397.71 ± 3.85^d^	71.02 ± 1.45^cd^	3.93 ± 0.00^e^	13.73 ± 0.15^e^	85.66 ± 3.05^bcd^
5	*L. plantarum*	422.63 ± 1.64^bc^	72.74 ± 1.49^abc^	4.00 ± 0.01^bcd^	14.16 ± 0.05^ab^	89.00 ± 2.00^ab^
6	*L. fermentum*	436.02 ± 3.41^a^	74.41 ± 1.06^ab^	4.01 ± 0.01^bc^	13.76 ± 0.05^de^	90.33 ± 2.51^a^
7	*L. acidophilus*	419.35 ± 0.69^c^	67.79 ± 1.29^e^	4.02 ± 0.01^ab^	14.06 ± 0.11^abc^	83.66 ± 1.52^cd^
8	*L. reuteri*	422.48 ± 3.35^c^	72.07 ± 1.59^bc^	3.97 ± 0.01^d^	14.26 ± 0.20^a^	85.33 ± 2.08^bcd^
9	*P. acidilactici*	400.33 ± 4.39^d^	72.89 ± 1.79^abc^	4.02 ± 0.00^ab^	14.03 ± 0.15^abcd^	82.66 ± 2.08^e^
10	*L. bulgaricus*	395.48 ± 3.06^d^	71.56 ± 2.21^bcd^	4.04 ± 0.00^a^	13.96 ± 0.15^bcde^	86.33 ± 2.08^abcd^
11	*L. paracasei*	423.85 ± 2.40^bc^	69.12 ± 1.47^de^	4.02 ± 0.02^ab^	14.03 ± 0.05^abcd^	87.66 ± 3.21^abc^

Values are from the mean ± standard deviation (SD), and different lowercase letters in the upper right corner of the values in the same column represent significant differences between the values (*p* < 0.05).

SOD is an important component of the antioxidant enzyme system in biological systems and has a strong antioxidant ability. SOD is not only naturally present in fruit and vegetable juices, it is also produced during the metabolism of LAB (20). After the fermentation of tomato juice, the SOD activity increased in all groups. Compared to the original solution producing, the *L. casei, L. plantarum, L. fermentum, L. paracasei* increased the SOD by 41.44, 35.63, 49.02, and 36.85 U/g, respectively. Explanations for these differences may include strain-specific functions, differences in growth rates and environments, and differences in carbon source utilization.

Xinjiang tomato fruits contain higher levels of LYC than those from other regions (21). LYC has been shown to have beneficial effects on human health. The LYC content produced by different strains varied. *P. pentosaceus, L. fermentum, P. acidilactici,. L. plantarum* was increased by 9.7, 8.2, 6.0, and 5.8%, respectively, compared to the unfermented samples. It may be due to fermentation hydrolysis and the destruction of the cell walls of tomato cells by LAB during the fermentation process. The destruction of the cells leads to a decrease in the pectin content and reduces the size of the tissue fragments, which reduces gravitational separation and promotes the release of LYC, thereby increasing the content (22).

The proportion of flavoring substances influences affects the sensation. The decrease in pH during fermentation may be due to the production of organic acids, and the absorption of a portion of glycogen by microorganisms, which leads to a decrease in TSS and destroys the flavor of the fermented juice. The highest sensory evaluation scores were given by *L. fermentum, L. plantarum, P. pentosaceus*, and *L. paracasei*, with scores of 90.33, 89.00, 88.33, and 87.66, respectively. For these results, the differences are due to the unique properties of each strain resulting in fermented samples with different acidic flavors and different organic acid compositions that give each sample a unique flavor. Besides, the volatile compound composition of the FTJ varies from strain to strain, and the senses will be different (23).

Combined fermentation of multiple strains can make up for the defects of other single strains in fermentation. Based on the three factors of high SOD, LYC, the best four strains, *P. pentosaceus, L. casei, L. plantarum*, and *L. fermentum* were selected for mixed fermentation. The optimum percentage of each strain was determined using a homogeneous design and the results are shown in Table 6, and the equation follows:


$$\begin{array}{l} Y=267.485+1284.208X_{1}-288.073{X_{2}}^{2}-2436.117{X_{3}}^{2}\\ \;-8973.864X_{1}X_{4}-6613.659X_{2}X_{3}\\ \;+5530.703X_{2}X_{4}+12747.912X_{3}X_{4} \end{array}$$

**Table 6:** Results of uniform design experiments.

No.	*P. pentosaceus* (%)	*L. casei* (%)	*L. plantarum* (%)	*L. fermentum* (%)	SOD activity (U/g)
1	40	13	19	21	430.10
2	41	15	24	19	449.83
3	42	17	18	17	426.90
4	43	19	23	15	409.10
5	44	21	17	13	432.90
6	45	12	22	22	423.10
7	46	14	16	20	380.13
8	47	16	21	18	415.46
9	48	18	15	16	416.33
10	49	20	20	14	420.10

R^2^ = 0.999 and *p* < 0.01, indicating that the equation is well-fitted and accurately predicts optimal conditions. In conclusion, the optimal inoculation rates of FTJ were predicted to be *P. pentosaceus* (53.79%), *L. casei* (13.17%), *L. plantarum* (19.87%), *L. fermentum* (13.17%), with a corresponding SOD activity of 525.76 U/g. Based on the optimal inoculation rate described above, the total strain inoculum of the four strains was maintained at 2 × 10^6^ CFU/mL and the TSS was maintained at 13.5^°^Brix. Fermentation for 22 h at 37°C. In the validation test, SOD activity reached 486.35 U/g.

### 3.2 Response surface experimental results and analysis of variance

Different fermentation environments have different effects on the results and to achieve the best product, it is necessary to optimize the fermentation conditions. According to the preliminary experiments showed no significant changes in pH and TSS content. Therefore, SOD and LYC were used as dual response values for the optimization of fermentation conditions.

**Figure 1:**
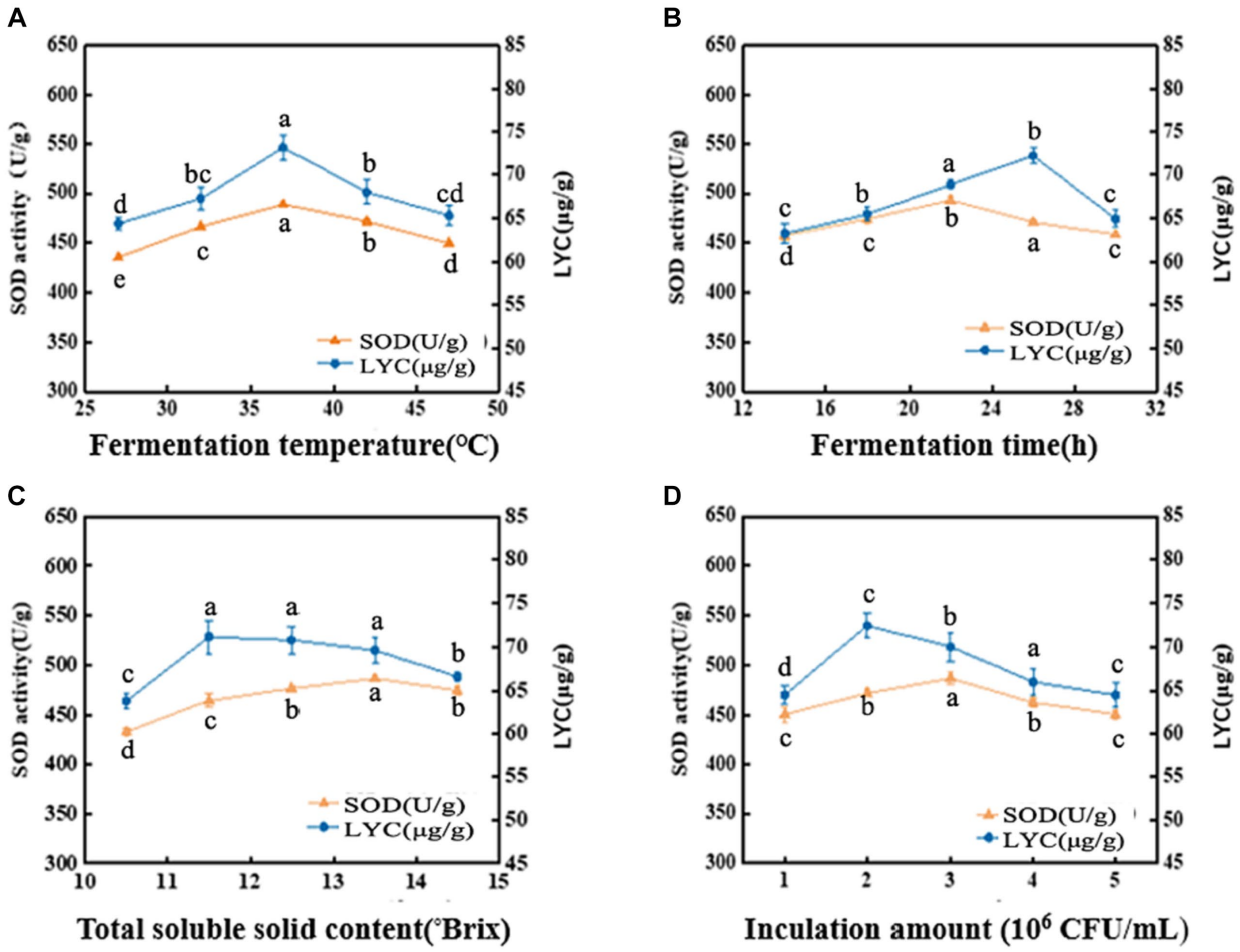
Effects of fermentation temperature **(A)**, fermentation time **(B)**, soluble solid content (°Brix) **(C)**, and inoculation amount **(D)** on SOD activity and LYC content. Different lowercase letters represent significant differences between values (*p* < 0.05).

The metabolic activity of LAB is affected by fermentation temperature, thereby affecting the fermentation quality (24). As shown in Figure 1A, the SOD activity is 488.78 U/g at 37°C, then decreased with increasing temperature. LYC also reached a maximum value of 73.13 μg/g at 37°C, after which it gradually decreased. The appropriate growth temperature for LAB is generally around 37°C, so the decrease in both SOD activity and LYC content may be due to over-high temperature. The intolerance of bacteria to heat can lead to a decrease in enzyme metabolism and activity (25). Hence, the optimal fermentation temperature was set at 37°C.

The SOD activity and LYC content were affected by the fermentation time as shown in Figure 1B. When the fermentation time is less than 14 h, the growth and reproduction of LAB were insufficient, metabolites were not accumulated enough, and the SOD activity and LYC content were low. If the time is too long, it will lead to a decrease in SOD activity and LYC content because insufficient nutrients will lead to an increase in the number of dead bacteria and an excessive accumulation of metabolic wastes. SOD activity increased rapidly between 14 h and 22 h and was 493.06 U/g at 22 h, afterwards decreased. The LYC reached a maximum value of 72.20 μg/g at 26 h and then decreased with increasing fermentation time. Therefore, the optimal fermentation time is 22–26 h.

As shown in Figure 1C, the SOD activity was (486.95 U/g) when the TSS content was adjusted to 13.5^°^Brix, and the LYC reached 71.14 μg/g at a TSS content of 11.5^°^Brix. From these results, we chose the range of 11.5^°^Brix-13.5^°^Brix.

From Figure 1D, it can be observed that When the strain number concentration was 10^6^ CFU/mL, the bacterial population was insufficient and the bacterial metabolic rate was slow. It induced a decrease of SOD activity and LYC. However, the values of the indicators increased with the increase of the inoculum. The LYC reached 72.43 μg/g when the strain number concentration was 2 × 10^6^ CFU/mL. the maximum SOD activity was 486.92 U/g. When the strain number con-centration was 3 × 10^6^ CFU/mL. If the inoculum exceeds 3 × 10^6^ CFU/mL, it may result in excessive fermentation, which can result in destabilization of terpenoids and loss of biological enzyme activity. As a result, the optimal inoculum amount ranges from 2 × 10^6^–3 × 10^6^ CFU/mL.

Experiments showed that four factors had effects on both SOD activity and LYC. The design and results of the Box–Behnken test were shown in Table 3. The regression equations are modeled below:


$$\begin{array}{l} Y1=503.10-0.1538A+10.48B-4.47C-9.97D\\ \;-8.49\mathrm{AB}+6.19\;\mathrm{AC}-0.0775\;\mathrm{AD}-6.53\;BC\\ \;+7.90BD+5.19CD-29.59A^{2}-12.11B^{2}\\ \;-31.52C^{2}-32.09D^{2} \end{array}$$

$$\begin{array}{l} Y2=77.25+1.03A\;0.7420B+1.02C+0.4004D\\ \;-0.5100\mathrm{AB}-1.02\;\mathrm{AC}-0.2075\;\mathrm{AD}-1.17\;BC\\ \;+0.5587BD-0.5475CD-3.18A^{2}-0.17B^{2}\\ \;-1.15C^{2}-0.9473D^{2} \end{array}$$

Anova and significance test for SOD activity and LYC. R^2^, adjusted R^2^, final results for flow rate flow %, and proper accuracy are shown in Table 7.

**Table 7:** Analysis of variance of SOD activity and LYC in FTJ.

Source	SOD activity (U/mL)		TFC (mg/mL)	
	*F*-value	*P*-value	*F*-value	*P*-value
Model	80.48	<0.0001^**^	24.34	<0.0001^**^
A-Temperature	0.0157	0.9020	31.69	<0.0001^**^
B-Fermentation time	105.46	<0.0001^**^	23.81	0.0002^**^
C-Inoculum amount	13.31	0.0026^**^	31.47	<0.0001^**^
D-Amount of added sugar	66.09	<0.0001^**^	4.80	0.0458^*^
AB	33.71	<0.0001^**^	5.48	0.0345^*^
AC	10.09	0.0067^**^	12.28	0.0035^**^
AD	0.0016	0.9688	0.5107	0.4866
BC	19.97	0.0005^**^	28.96	<0.0001^**^
BD	29.24	<0.0001^**^	6.58	0.0224^*^
CD	7.09	0.0185^*^	3.56	0.0803^*^
A^2^	373.83	<0.0001^**^	194.80	<0.0001^**^
B ^2^	197.96	<0.0001^**^	68.97	<0.0001^**^
C ^2^	424.22	<0.0001^**^	25.27	0.0002^**^
D^2^	439.56	<0.0001^**^	17.26	0.0010^**^
Lack of Fit	0.6268	0.7502^ns^	1.39	0.4013^ns^
R ^2^	0.9877		0.9605	
Adj. R ^2^	0.9755		0.9211	
Pred. R^2^	0.9494		0.8097	
Adeq precision	26.6468		16.3740	
C.V.%	0.8736		0.7804	

*p < 0.05, **p < 0.01.

**Figure 2:**
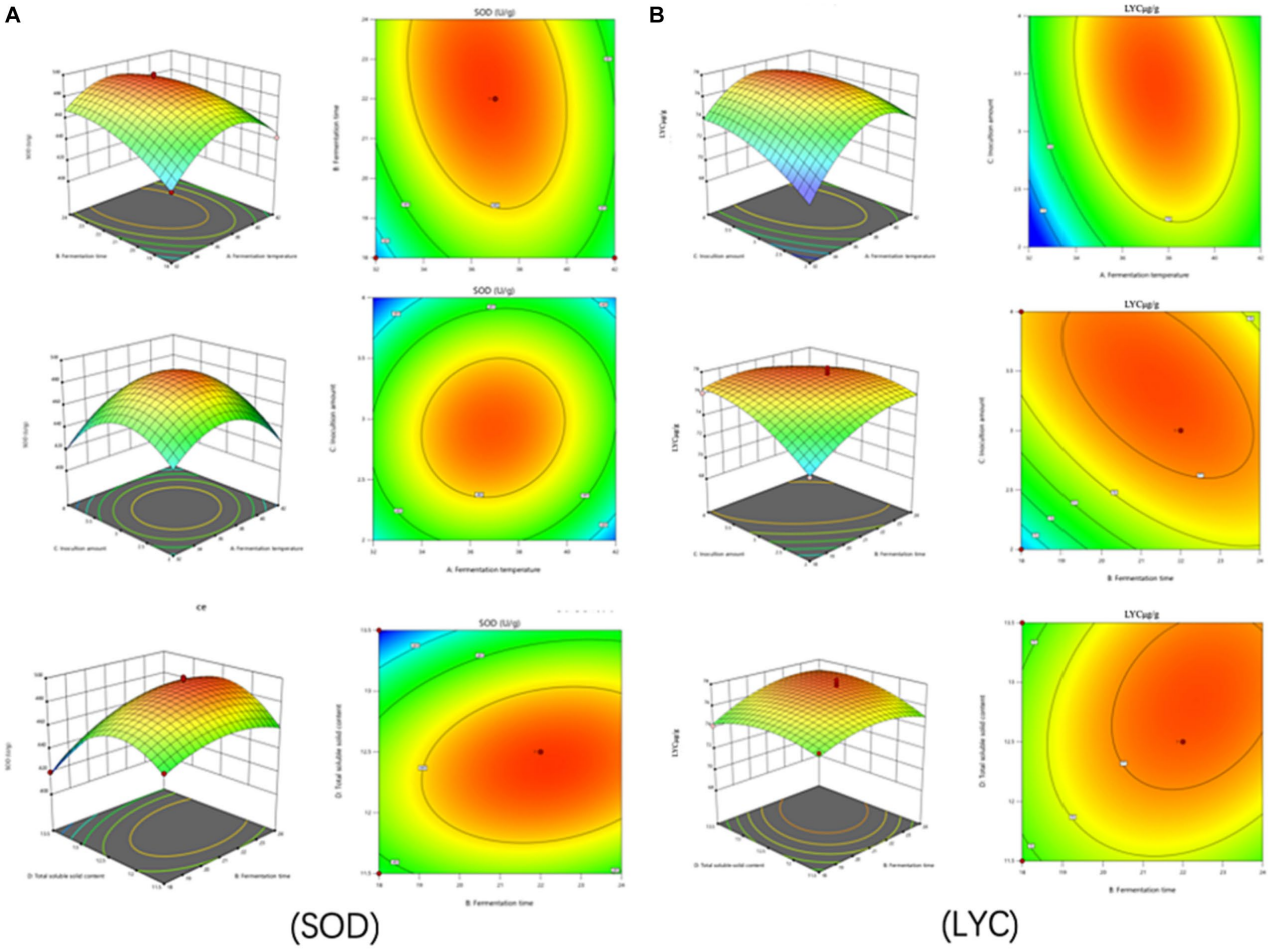
Effects of interaction of various factors on **(A)** (SOD) activity and **(B)** (LYC) concentration of FTJ.

The test of significance of the coefficients of the model regression equation for Y1 (Figure 2A) shows that the linear coefficients (B, D, AB, AD, BD, BC, A^2^, B^2^, C^2^, and D^2^) are highly significant and the rest of the terms are not significant. The test of significance of coefficients of regression equation of the Y2 model (Figure 2B) shows that linear coefficients (A, B, C, AC, BD, A^2^, B^2^, C^2^, and D^2^) are highly significant and the rest of the terms are not significant (*p* < 0.01).

As can be seen from Table 7, the correlation coefficient R^2^ value for SOD activity was 0.9877 and for LYC it was 0.9605, indicating that the model can be used for the analysis and prediction of the fermentation process. By predicting the regression model, the best treatment for FTJ was obtained as follows: fermentation temperature of 37.09°C, LAB inoculum of 2.95 × 10^6^ CFU/mL, fermentation time of 22.20 h, and TSS of 12.45^°^Brix. In these conditions, the predicted value of FTJ SOD activity was 505.79 U/g, and the predicted value of LYC was 77.33 μg/g.

After comprehensive consideration, the optimal conditions were modified to a fermentation time of 22 h, fermentation temperature of 37°C, LAB inoculum of 3 × 10^6^ CFU/mL, and sugar addition of 12.5^°^Brix. Under these conditions, we conducted experiments and verified that the SOD activity of the tomato enzyme was 496. 67 U/g and the LYC content was 77.12 μg/g.

### 3.3 Multivariate statistical analysis

**Figure 3:**
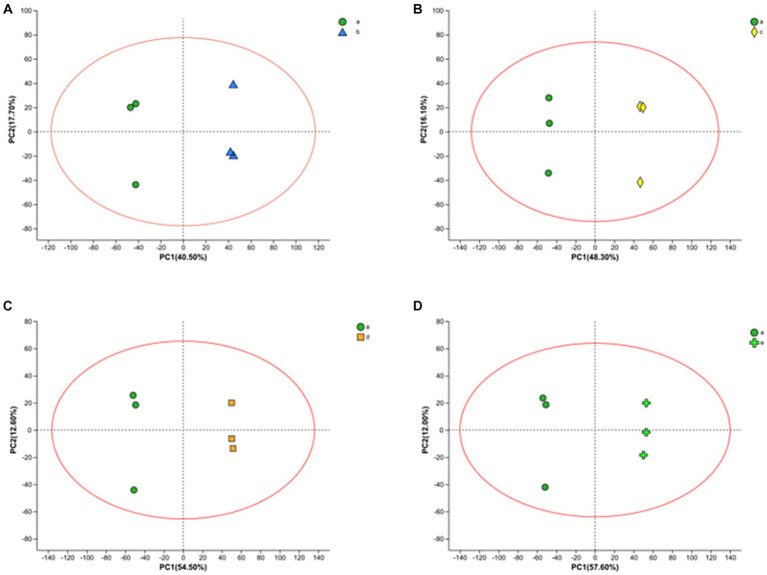
PCA score plots of FTJ during different multi-strain fermentation stages [**(A)** 0–10 h; **(B)** 0–16 h; **(C)** 0–20 h; **(D)** 0–22 h].

LC–MS technique was used to characterize the metabolites in the fermentation broth to gain insight into the metabolites at different time stages during the fermentation process. A total of 1867 ionic metabolite signatures were generated at five stages, A, B, C, D, and E, during the fermentation process, including 1,020 positive ion patterns and 847 negative ion patterns. The metabolites were further analyzed by applying PCA and OPLS-DA models to the processed metabolite lists, which can effectively reduce the data dimensionality and improve the interpretability and validity of the data. PCA responds to the overall temporal and within-sample variability, reveals the distribution trends among various samples, and identifies possible discrete dispersal points, which can intuitively reflect the similarities or differences among the samples. As shown in Figure 3, fermentation samples at 0 h–10 h, 0 h–16 h, 0 h–20 h, and 0 h–22 h were separated by PCA principal component differentiation. All samples and QCs were within the 95% confidence interval, indicating good stability and reproducibility of the experiment.

**Figure 4:**
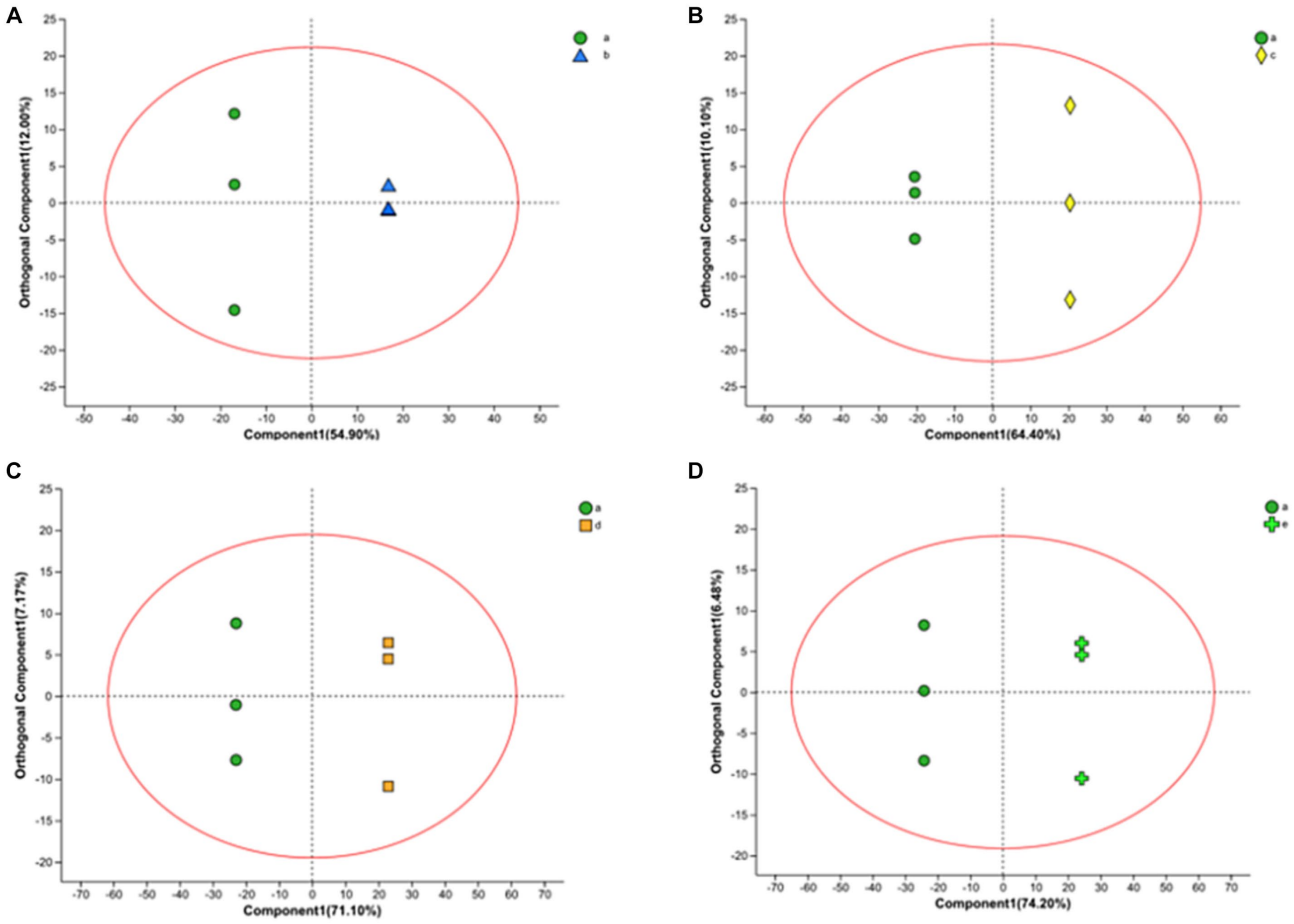
OPLS-DA in positive ion mode yields scatter plot [**(A)** 0–10 h; **(B)** 0–16 h; **(C)** 0–20 h; **(D)** 0–22 h].

In this work, we also analyzed the metabolite variation using the OPLS-DA model. OPLS-DA is an orthogonal correction that filters out uncorrelated orthogonal signals, ensuring a valid and more reliable model. In the model, there were four groups, and their OPLS-DA score plots (Figure 4) showed excellent model parameters (0 h–10 h: R^2^Y = 1, Q2 = 0.963; 0 h–16 h: R^2^Y = 1, Q^2^ = 0.983; 0 h–20 h: R^2^Y = 1, Q^2^ = 0.990; 0 h–22 h, R^2^Y = 1, Q^2^ = 0.993). In cross-validation and response alignment, tests showed that no overfitting occurred in the OPLS-DA model (Supplementary Figure S1). Therefore, the OPLS-DA model is valid with high predictive power and can be used to explore metabolic differences at different stages during the fermentation of FTJ.

### 3.4 Screening for differential metabolites

#### 3.4.1 Determination of differential metabolites

The projection of the first principal component (VIP) from the OPLS-DA analysis was utilized to assess the strength and interpretability of the effect of expression patterns between groups. Typically, differential metabolites were identified as VIP > 1. In this study, the metabolites with VIP > 1 and *p* < 0.05 were selected to screen four groups (0–10 h, 0–16 h, 0–20 h, and 0–22 h) in a positive–negative ion mode. Overall, we identified 168 differential metabolites throughout the fermentation process, 75 differential metabolites during 0–10 h, 111 differential metabolites during 0–16 h, 136 differential metabolites during 0–20 h, and 166 differential metabolites during 0–22 h (Supplementary Table S1). Among these differential metabolites, organic acids, amino acids, and their derivatives, etc. accumulated mainly due to lactobacilli metabolism, whereas there were lipids and lipid-like molecules mainly consumed due to fermentation, suggesting that carbohydrates, certain amino acids, and lipids were the main precursors of the metabolites.

**Figure 5:**
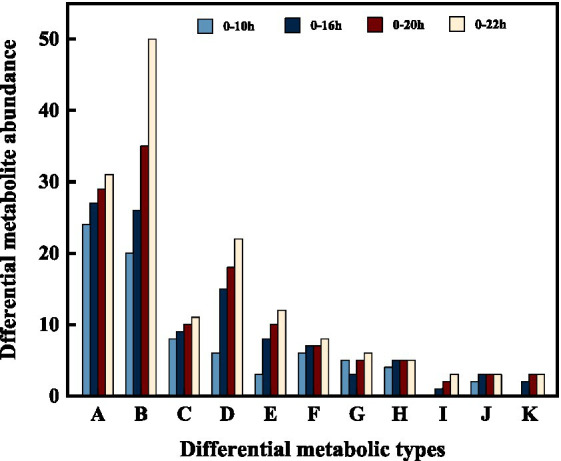
Changes of differential metabolites in FTJ during different multi-strain fermentation stages [A: Peptides; B: amino acid; C: heterocyclic compounds; D: phospholipid; E: carbohydrate; F: organic acid; G: steroid; H: amine; I: terpene; J: sulfur-containing compounds; K: fatty acid].

**Figure 6:**
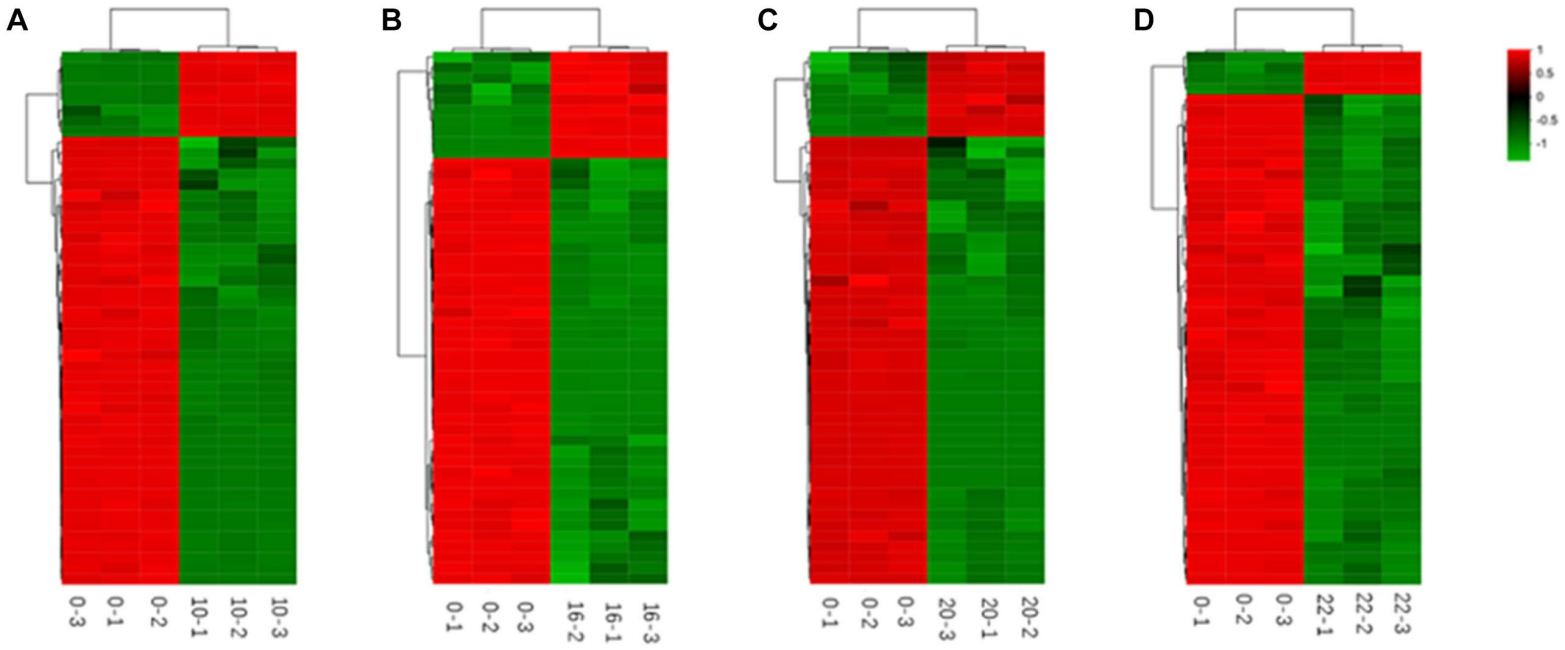
Heat maps of metabolites differing by fermentation times [**(A)** 0–10 h; **(B)** 0–16 h; **(C)** 0–20 h; **(D)** 0–22 h].

In addition, we categorized and ranked the major differential metabolites (Figure 5) to gain more insight into the changes in differential metabolites during FTJ. The results showed that the major different metabolites during FTJ were peptides, amino acids, and carbohydrates. These metabolites have different metabolic profiles in different fermentation stages. It is noteworthy that the metabolic properties of the primary metabolites showed relative differences at different stages of the fermentation process. The species and relative abundance of the difference metabolites were the primary ways in which they were expressed. We also constructed a hierarchical cluster analysis (HCA) (Figure 6), the findings of which were consistent with the differential metabolites.

#### 3.4.2 Dynamics of carbohydrates, phospholipids, organic acids, and terpenoids during fermentation

**Figure 7:**
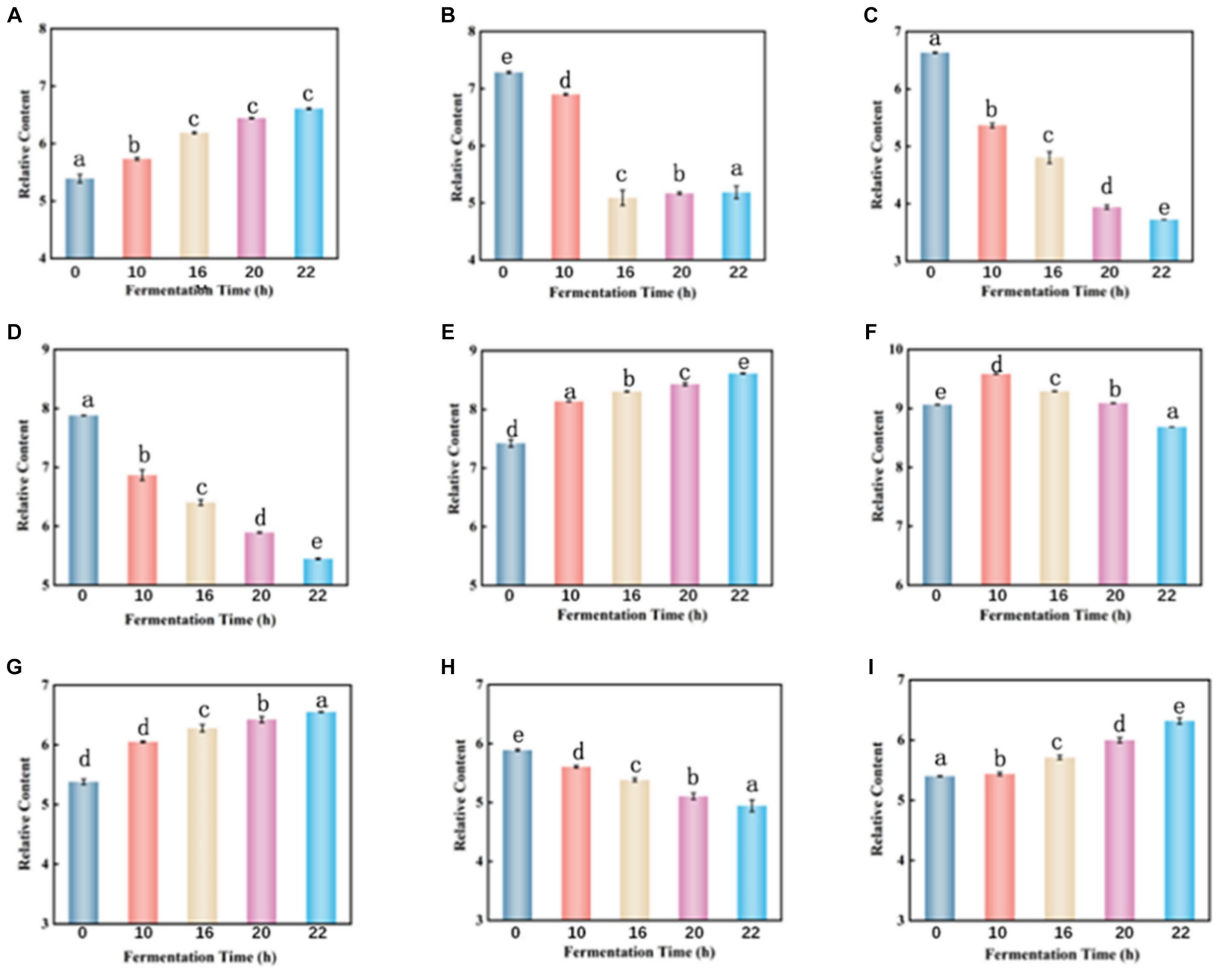
Dynamic changes of key differential metabolites in FTJ during different multi-strain fermentation stages [**(A)** Galactitol; **(B)** cytarabine; **(C)** uridine; **(D)** malic acid; **(E)** lactic acid; **(F)** citric acid; **(G)** 16-deacetylgeyerline; **(H)** (+)-Royleanone; **(I)** artecanin]. Different lowercase letters represent significant differences between values (*p* < 0.05).

Throughout the fermentation process, the total amount of carbohydrate matter decreased from an initial 1118.6 to 1109.6. Galactitol is commonly used as a low-calorie sweetener and can be a potential component of biobased chemicals. During fermentation, the content of galactitol increased from an initial 5.389 to 6.607 (Figure 7A), which may be related to the hydrolysis of sucrose, and other complex polysaccharides in tomato enzymes (26). The content of cytarabine decreased by 29% (Figure 7B), which may be due to enzymatic deglycosylation. Uridine decreased by 44% (Figure 7C) due to the use of lactobacillus fermentation which requires carbohydrates as an energy source for growth (19).

Malic acid is highest in unfermented tomato juice and decreases with longer fermentation time (Figure 7D). LAB can metabolize malic acid using malic acid lactase, which decarboxylates it to lactic acid using NAD^+^ and Mn2^+^-dependent malic acid lactase (27–29). However, *L. casei* can also utilize malic enzymes to break down malate into pyruvate, allowing them to use malate as a carbon source for growth (30). Lactic acid is the primary organic acid produced during LAB fermentation. Its content slightly increases with the duration of fermentation (Figure 7E). Lactic acid is produced by LAB through sugar metabolism, but this is not the only way LAB produces this metabolite. In fact, polyols (e.g., glycerol) or acids (e.g., malic acid) may also be metabolized to produce lactic acid (31). Lactic acid and typically low pH can damage cell walls and membranes, altering membrane potential and active transport (32), leading to energy depletion and cell death. Therefore, the preservation of fermented juices is favored due to the high lactic acid content during fermentation. Citric acid is known to be a key intermediate in the tricarboxylic acid (TCA) cycle (33). Therefore, citric acid increased at the beginning of fermentation but decreased thereafter (Figure 7F). Citric acid can be transformed by citrate lyase in LAB to oxaloacetate and acetic acid, whereas oxaloacetate can be converted to pyruvate, which can then be converted by LAB to the flavoring molecule 3-hydroxybutanone (34).

Terpenoids are a diverse group of plant secondary metabolites consisting of several isoprene units (35). These bioactive compounds confer a wide range of biological activities such as anticancer, antiallergic, antimicrobial, and antioxidant activities (36, 37). When the fermentation process was completed, 99% of the total terpenoid content was retained, with 1.21-fold up-regulation of 16-deacetylgairin and 1.22- go down-regulation of (+)-Royleanone (Figures 7G–I). Artemisinin is a sesquiterpene endoperoxide with potent antimalarial properties (38). Artemisinin was also slightly elevated during fermentation. It indicated that terpenoids were extracted from the berries during the fermentation process (39).

Phospholipids are major structural components of cell membranes and are essential for various cellular processes (40). In previous studies, it was shown that phosphatidylserine (PS) and phosphatidylethanolamine (PE) are high quality phospholipids. Studies have shown that the combined use of PS and PE lipids can be effective in the treatment of some cancers (41). The quantitative results of PS, PE, PC, PG, and PI were shown in Table 8. The total amount of all of them decreased to different degrees after fermentation. PC content decreased from 41.138 to 35.755 and PE content decreased from 56.543 to 46.964. Although the intensity of PS was lower compared to the others, it also decreased after fermentation with PS content decreasing from 11.119 to 9.186 (Table 8). The results showed that most of these species contain unsaturated fatty acyl fractions, including oleic (18:1), linoleic (18:2), and linolenic (18:3) acid chains. Specifically, esterified polyunsaturated 18:2 and 18:3 chains predominated. This result suggests that phospholipids containing 18:2 and 18:3 chain acyl groups underwent degradation in microorganisms during fermentation.

**Table 8:** Dynamic changes of key phosphatidylinositol in FTJ during different multi-strain fermentation stages.

Name	Marker compound	Ion model	0	10	16	20	22
PS	LysoPS(18:2(9Z,12Z)/0:0)	Pos	6.4836	5.8108	5.1117	4.7881	4.2589
	LysoPS(18:1(9Z)/0:0)	Neg	5.5759	5.6997	5.7075	5.7909	5.7958
PE	LysoPE(18:2(9Z,12Z)/0:0)	Pos	7.5012	6.9392	6.4898	6.3124	6.2461
	LysoPE(16:0/0:0)	Pos	6.0563	4.9907	3.6795	3.6507	3.6233
	PE(14:0/14:1(9Z))	Pos	5.7653	5.7884	5.8141	5.8289	5.8220
	LysoPE(0:0/22:6(4Z,7Z,10Z,13Z,16Z,19Z))	Neg	5.5831	5.5824	5.5681	5.5908	5.5807
	PE(16:0/0:0)	Neg	5.7182	5.1365	4.8070	4.7156	4.7254
	LysoPE(0:0/18:3(9Z,12Z,15Z))	Neg	6.4544	5.8502	5.3014	4.8768	4.6092
	LysoPE(18:3(9Z,12Z,15Z)/0:0)	Neg	6.1163	5.1672	4.4981	4.2480	3.9381
	LysoPE(20:5(5Z,8Z,11Z,14Z,17Z)/0:0)	Neg	5.8900	5.9239	5.9135	5.9310	5.9208
	PE(18:2/0:0)	Neg	7.4580	6.9304	6.6129	6.4954	6.4979
PC	LysoPC(18:2(9Z,12Z)/0:0)	Pos	7.9526	7.0109	6.4531	6.2886	6.0624
	PC(18:1(12Z)2OH(9,10)/2:0)	Pos	2.9471	5.6862	5.7116	5.8288	6.0596
	LysoPC(18:1(11Z)/0:0)	Pos	6.6764	6.0620	5.6456	5.3707	5.1022
	LysoPC(0:0/18:2(9Z,12Z))	Pos	5.7736	4.7333	3.6030	3.6329	3.6078
	LysoPC(18:3(6Z,9Z,12Z)/0:0)	Pos	6.7973	5.5602	4.8267	4.7051	4.3512
	LysoPC(14:1(9Z)/0:0)	Neg	5.4462	5.3959	5.3507	5.3351	5.3038
	PC(17:2(9Z,12Z)/0:0)	Neg	5.5451	5.7233	5.3914	5.3273	5.2678
PG	PG(16:0/0:0)	Neg	6.3946	5.2538	6.0129	5.8495	5.6615
	LysoPG(16:0/0:0)	Neg	5.6216	5.5076	5.2768	5.0261	4.8116
	LysoPG(18:2(9Z,12Z)/0:0)	Neg	5.7499	5.3527	4.9343	4.6437	4.2765
PI	PI(20:5(5Z,8Z,11Z,14Z,17Z)/0:0)	Pos	6.3779	6.1558	5.8257	5.4085	5.0213
	LysoPI(18:2(9Z,12Z)/0:0)	Neg	7.7322	7.5037	7.2196	6.9157	6.6915
	LysoPI(16:0/0:0)	Neg	7.0630	6.4191	5.8240	5.4846	5.2309

Lipidomics analysis was conducted according to previous studies (42), and we predicted that the phospholipids in this study may follow a similar degradation pathway. In the presence of hydrolytic enzymes from the microbial community, phospholipids undergo a lipolytic reaction that releases free fatty acids, namely phospholipids PE and PC, and simultaneously generates solubilized phospholipids. These changes could be the primary cause of the decline in phospholipid content. Various fatty acids elongate fatty acids, leading to the production of very long-chain fatty acids with a wide range of chain lengths (42). Fatty acids can be converted to hydroxy fatty acids through a series of microbial fatty acid hydroxylase reactions. Hydroxy fatty acids can be further transformed into hydroxy fatty acid branched-chain fatty acid esters by acylation reactions. Moreover, hydroxy fatty acids may be integrated into sphingolipids such as ceramides (43), thus contributing to sphingolipid accumulation.

## 4 Conclusion

In this study, the suitability of 10 probiotic strains for tomato juice fermentation was investigated and 4 of the most suitable strains were selected. The FTJ process was optimized using a homogeneous design and RSM. The optimal fermentation conditions for FTJ fermentation were determined as follows: fermentation time of 22 h, TSS content of 13^°^Brix, fermentation temperature of 37°C, and inoculum concentration of 3 × 10^6^ CFU/mL.

We analyzed key differential metabolites of fermented tomato juice using UHPLC-QE-MS/MS and identified 168 different metabolites: including carbohydrates, phospholipids, organic acids, and terpenes. Changes in carbohydrates such as galactitol, aglycone, and uridine were related to the hydrolysis of sucrose and polysaccharides in the fermented tomato juice and their need for LAB-fermentation as an energy source for growth. The changes in phospholipids (PS, PE, PC, PG, PI) are since most of these species contain unsaturated fatty acyl portions and they can undergo degradation during microbial fermentation. Organic acids (malic, citric, lactic) can be altered because each has a different metabolic pathway. During fermentation, 16-deacetylgairin, (+)-Royleanone, and artemisinin have different degrees of variation with fermentation time due to the terpenoids that can be extracted from the stock solution. Due to the limitations of the specific strains and experimental conditions used in this study, there may be some deficiencies in general applicability, and further studies involving a wider range of strains and fermentation conditions are needed to improve the applicability of the results. All in all, this study provides further insight into the fermentation process of tomato juice and the dynamic changes of metabolites during fermentation and lays the foundation for the study of fruit and vegetable fermentation.

## Data availability statement

The original contributions presented in the study are included in the article/Supplementary material, further inquiries can be directed to the corresponding authors.

## Author contributions

LZ: Writing – original draft. RM: Writing – review & editing. JH: Writing – review & editing. LW: Writing – review & editing. YM: Writing – review & editing. BL: Writing – review & editing. HZ: Writing – review & editing. KC: Writing – review & editing. AA: Writing – review & editing.

## Footnotes

^1^FAO, 2020, https://www.fao.org/faostat/en/-data/QCL, accessed date 25 April 2022.
