# Corrigendum: Optimization of tomato (*Solanum lycopersicum* L.) juice fermentation process and analysis of its metabolites during fermentation

**DOI:** 10.3389/fnut.2024.1423909

**Published:** 2024-05-17

**Authors:** Lei Zhao, Ruxianguli Maimaitiyiming, Jingyang Hong, Liang Wang, Ying Mu, Bingze Liu, Huimin Zhang, Keping Chen, Aihemaitijiang Aihaiti

**Affiliations:** ^1^School of Life Science and Technology, Xinjiang University, Urumqi, China; ^2^Xinjiang Huize Food Limited Liability Company, Urumqi, China

**Keywords:** fermentation, tomato juice, superoxide dismutase, response surface methodology, UHPLC-QE-MS/MS

In the published article, Supplementary Table 1 and Supplementary Figure 1 were mistakenly not included in the publication. The missing material has been published in the original article.

**Supplementary Figure 1**

Plot of cross-validation and alternate testing of OPLS-DA model (ESI+).

**Supplementary Table 1**

Detailed list of metabolites differing between groups.

The authors apologize for this error and state that this does not change the scientific conclusions of the article in any way. The original article has been updated.

