# Supplementary material for: Optimization of tomato (Solanum lycopersicum L.) juice fermentation process and analysis of its metabolites during fermentation
Source: Front Nutr. 2024 Feb 1;11:1344117. doi: 10.3389/fnut.2024.1344117 (PMC10868405; doi:10.3389/fnut.2024.1344117)
Supplement: Supplementary file 1 [file Image_1.pdf]

Textual notes on supplementary materials

Supplementary Materials: Figure S1: Plot of cross-validation and alternate testing of OPLS-DA model (ESI+).

Response sequencing test (RPT) of OPLS-DA model

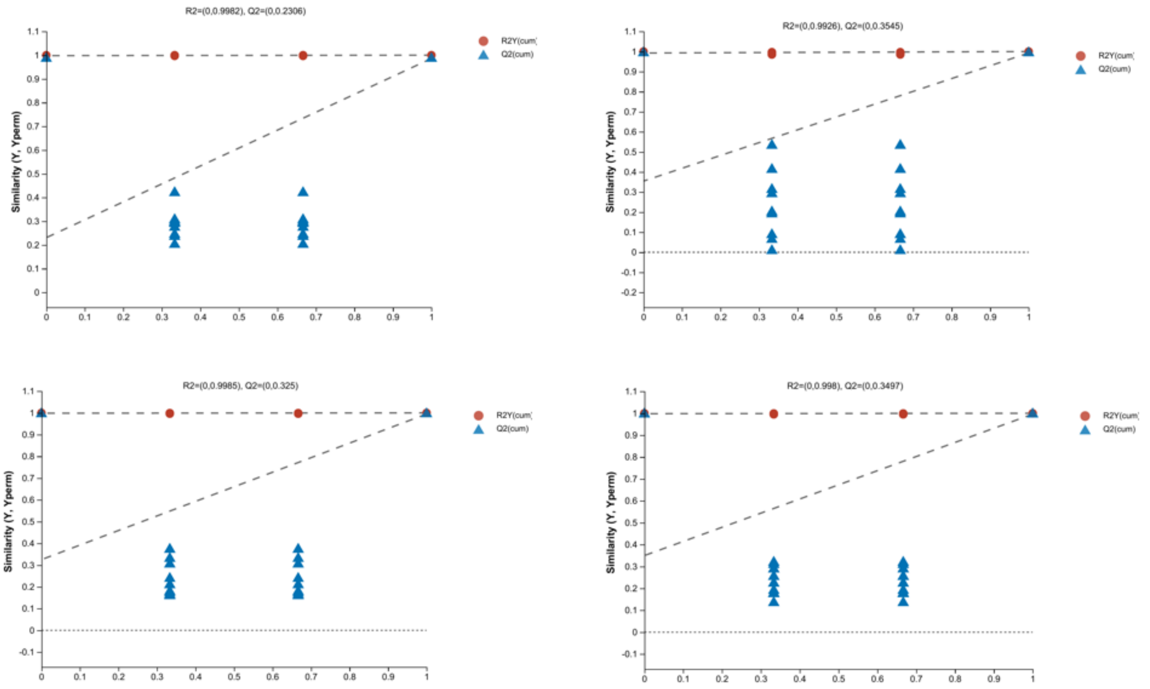

Supplementary Materials: Table S1: Detailed list of metabolites differing between groups.
